# Assessing synovitis in the hands in patients with rheumatoid arthritis by ultrasound: an agreement study exploring the most inflammatory active side from two Norwegian trials

**DOI:** 10.1186/s13075-019-1930-y

**Published:** 2019-07-05

**Authors:** Lene Terslev, Robin Christensen, Anna-Birgitte Aga, Joe Sexton, Espen A. Haavardsholm, Hilde B. Hammer

**Affiliations:** 1 Center for Rheumatology and Spine Diseases, Rigshospitalet, Valdemar Hansens vej 17, Glostrup, DK-2600 Copenhagen, Denmark; 2 Musculoskeletal Statistics Unit, The Parker Institute, Bispebjerg and Frederiksberg Hospital, Frederiksberg, Denmark; 3 Department of Clinical Research, Research Unit of Rheumatology, University of Southern Denmark, Odense University Hospital, Odense, Denmark; 4 Department of Rheumatology, Diakonhjemmet Hospital, Oslo, Norway; 5 Department of Health Management and Health Economics, University of Oslo, Oslo, Norway

**Keywords:** Synovitis, Rheumatoid arthritis, Ultrasound, Doppler, Inflammation, Dominant, Hand

## Abstract

**Objective:**

To assess if the right hand, the dominant hand, or the hand with more clinically swollen joints (SwJ) is per se the most inflamed and exhibits the greatest change during treatment and hence preferred for unilateral scoring of synovitis by ultrasound in rheumatoid arthritis (RA) patients.

**Methods:**

Using data from two previously published Norwegian RA patient cohorts initiating treatment, bilateral metacarpophalangeal joint 1–5, proximal phalangeal joint 2+3, and wrists were evaluated by ultrasound. Using a 0–3 scoring system a grey-scale (GS), power Doppler (PD) and global synovitis score (GLOESS) was calculated for each hand (0–30). For precision, a difference of < ± 3 in sum score was pre-specified as indicating clinically insignificant difference in inflammatory activity for all three scores.

**Results:**

Four hundred thirty-seven RA patients were included. Baseline ultrasound inflammation was statistically significantly higher in hands with more vs fewer SwJ ([mean difference, 95%CI] GS sum score 2.21[1.30 to 3.12], PD sum score 1.70 [0.94 to 2.47] and GLOESS 2.31[1.36 to 3.26]) and also exhibited significantly more change for all sum scores at 3 months follow-up (GS sum score 1.34 [0.60 to 2.08], PD sum score 1.17 [0.44 to 1.91], and GLOESS 1.43 [0.63 to 2.22]). No such differences were found between the dominant and the non-dominant or the right and the left hands at any time points.

**Conclusion:**

The hand with clinically more SwJ is statistically more inflammatory active according to GS, Doppler, and GLOESS sum scores, exhibits a change during treatment, and is potentially the best choice for unilateral scoring systems.

**Electronic supplementary material:**

The online version of this article (10.1186/s13075-019-1930-y) contains supplementary material, which is available to authorized users.

## Background

Ultrasound has been validated as an outcome measurement tool for assessing synovitis by grey-scale (GS) and Doppler in rheumatoid arthritis (RA) [1, 2]. Though recent publications have indicated that ultrasound examination of all RA patients—if offered a very tight clinical control—may not be necessary for obtaining clinical remission [3, 4], there are several situations in the clinic where ultrasound has a role in monitoring synovitis as an indicator of disease activity. Furthermore, ultrasound is used in clinical trials for assessing treatment response and remission. The components defining synovitis (synovial hypertrophy and hyperemia) are usually scored separately using a semi-quantitative scoring system (0–3) for indicating the grade of severity of the individual synovitis component [5]. In the recently published OMERACT-EULAR combined scoring system [1], it is suggested to apply the highest score of the two components as the final score for the joint. Scoring GS synovial hypertrophy and Doppler activity separately or in combination is sensitive to change during treatment both on joint and patient level—for the latter using a sum score [6]. When applied in a clinical trial context, several reduced joint sets, ranging from 6 to 12 joints, have been proposed for scoring synovitis over the last years [7–11]. These reduced joint sets evaluate synovitis either unilaterally or bilaterally, aiming at maintaining as much information about the inflammatory load of the patient as seen in the more elaborate and time-consuming joint evaluations (32–78 joints) [12, 13].

Scoring synovitis unilaterally will by far reduce the examination time [9], and the unilateral 7-joint count by Backhaus et al. [7] is often used in trials. In unilateral scoring systems, the suggestion is to choose either the dominant side [7, 8] or the clinically most affected side defined as the hand with the most tender and/or swollen joints [9, 14, 15]. The dominant side is also commonly the chosen side in magnetic resonance imaging (MRI), where only one side can be evaluated at a time and is based on data showing that the dominant hand has more erosions on X-ray than the non-dominant hand both at baseline and follow-up [16, 17]. However, there is no evidence per se to support the dominant hand as being more inflammation prone than the non-dominant hand at time of treatment initiation. Because of the lack of evidence-based guidance, we decided to assess which selection is likely to identify the more inflamed hand, a research-on-research project that could provide information about RA presentation of inflammation and could potentially serve as a guide for the “default option” in unilateral scoring systems among ultrasonographers in rheumatology and thus ensure better homogeneity among future studies and clinical reports.

The primary aim of this study was to investigate if one hand is always more inflamed than its counterpart as this would have implications for a unilateral scoring system applied in the clinic and for the application in clinical trials. This was assessed by evaluating if the right hand, the dominant hand (as indicated by MRI), or the hand with clinically more swollen joints is more likely to have higher ultrasound measured inflammation than its counterpart at the time of inclusion, as well as for changes during treatment from baseline to 3 months follow-up.

## Patients and methods

This research-on-research project was performed according to a pre-defined Statistical Analysis Plan that was uploaded in advance (see Additional file 1). The study was designed to identify at baseline the hand with most ultrasound-verified inflammation and hence most appropriate for potential unilateral ultrasound assessment, and further to investigate the change in inflammation of this selection at 3 months follow-up. Hence, the follow-up study was not about assessing treatment effect over time, but about evaluating the ability of the inflammation to change.

This study is a result of secondary analyses based on two independent ultrasound datasets including an early RA cohort (ARCTIC trial; ClinicalTrials.gov database [NCT01581294]) and an established RA cohort (ULRABIT trial; Anzctr.org.au database [ACTRN12610000284066]) initiating conventional synthetic disease-modifying anti-rheumatic drugs (DMARDs) in the early cohort and initiating or switching biological DMARD (bDMARD) in the established cohort. From the ARCTIC trial (recruited between September 2010 and April 2013), 238 patients with early RA were included, and in the ULRABIT trial (recruited from January 2010 to June 2013), 212 patients with established RA were included.

### Ultrasonography assessments

In these analyses, the hands were used as model evaluating metacarpophalangeal joint (MCP) 1–5, proximal interphalangeal joint (PIP) 2 and 3, and the wrist. The selection of PIP joints was based on the study of Backhaus et al. [7]. In the original ULRABIT trial, extensive ultrasound examinations were performed by a single experienced sonographer, and in the ARCTIC trial, by several experienced sonographers who had been trained in annual ultrasound workshops with both static and dynamic hands-on exercises to calibrate readers. In both studies, synovitis was defined hypoechoic synovial hypertrophy with or without Doppler activity including all hypoechogenic tissue inside the joint capsule as synovitis. Though effusion may be hypoechoic, it is mostly anechoic and a rare finding in RA wrists and finger joints in contrast to knees and MTP joints and therefore not likely to influence the current analysis. Hence for the hands, the definition for synovitis is in line with the modified OMERACT definition [1].

A 0–3 semiquantitative scoring system for both GS and power Doppler findings was applied in each of the following 36 joints: MCP 1–5, PIP 2–3, radiocarpal, midcarpal, distal radioulnar, elbow, knee, talocrural, and metatarso-phalangeal (MTP) 1–5 bilaterally using the ultrasound atlas by Hammer et al. as a reference which had demonstrated a high inter- and intra-observer reliability [18]. In the ULRABIT study, a Siemens Antares Sonoline machine was used (Siemens Medical solutions, Mountain view, CA, USA) with a linear probe (5–13 MHz and setting at 11.4 MHz) and unchanged Doppler settings optimized for slow flow [19]. In the ARTIC study, the same Siemen Antares Sonoline machines or GE Logiq E9’s (GE Medical Systems Ultrasound and Primary Care Diagnostics, Wauwatose, WI, USA) were used at baseline and follow-up in the participating centers (11 hospitals), all with linear probes and identical unchanged Doppler settings optimized for slow flow [19].

In the current analyses, both GS and power Doppler sum scores, as well as the OMERACT-EULAR combined score (GLOESS) [1], were calculated for each hand (for all sum scores, the range is 0–30). The highest score of GS and PD was defined as the GLOESS score for the individual joint.

A difference of less than 3 in ultrasound sum scores between the hands was perceived by the authors to be clinically insignificant based on expert opinion and was prespecified in the statistical analysis plan (Additional file 1).

### Laboratory and clinical examinations

For both trials, each visit included laboratory assessments of erythrocyte sedimentation rate (ESR) and C-reactive protein (CRP, mg/L) and 0–100-mm visual analogue scales (VAS) for assessor’s and patient’s global assessments of disease activity. In the ARTIC study, anticyclic citrullinated peptide (anti-CCP) and rheumatoid factor (RF) was assessed at baseline whereas this had been assessed prior to the study for the ULRABIT cohort. For the early RA cohort, 44 swollen joint counts (44 SJC) and Ritchie Articular Index were performed, while 28 swollen and tender joint counts (28 SJC and 28 TJC, respectively) were performed in the established RA cohort. The Disease Activity Score (DAS) was calculated in the early RA cohort, whereas the DAS28 was calculated in the patients with established RA; both scores were based on ESR.

### Statistical methods

In this agreement study, our objective was to see whether various approaches showed sufficient agreement to be applied interchangeably. Our initial approach was based on the Bland-Altman graphical techniques [20] to visually present a potential pattern between left versus right hand as the plot of difference against the mean of the two. This step also included a plot of the data and draw the line of equality on which all points would lie if the two ultrasound measures (left versus right) gave exactly the same reading every time. This scatter plot helps the eye in gauging the degree of agreement between measurements. Also, we use a plot of difference (right versus left) against mean which allows us to investigate any possible relationship between the measurement error and the true value. By calculating mean difference (∆: left − right) and the standard deviation of the differences (SD∆), we would expect most of the differences to lie between ∆ ± 1.96 × SD∆ if the differences are normally distributed where 95% of differences will lie between these limits [20].

According to our prespecified statistical analysis plan (Additional file 1), we wanted to evaluate whether there is a potential difference in ultrasound inflammation or two contextual factors: dominant hand and the hand with most swollen joints, respectively. Using SAS for Mixed Models (PROC MIXED), the similarity between measures was tested. The mixed effect model took into account any differences in patient characteristics (e.g., disease duration) between groups by including a fixed factor for cohort (i.e., adjusting for the “cohort effect”). The least squares mean values and the difference between them are reported based on a statistical model including a factor for the specific analysis (i.e., dominant/right/swollen), and trial (ARCTIC and ULRABIT, respectively) as fixed effects, with the patient-ID as a random effect. We a priori defined a reasonable equivalence margin between measures, to be a 95% confidence interval around the observed paired mean difference: − 2.99 to + 2.99 (Additional file 1).

## Results

Baseline and follow-up demographics for the combined cohort as well as for the sub-cohorts are shown in Table 1. The total cohort consisted of 442 patients and comprised the ARCTIC cohort with 230 DMARD naïve patients with early RA with indication for methotrexate (as 8 of the original 238 patients were subsequently excluded from the study as they did not receive treatment) and the ULRABIT cohort with 212 established RA patients with indication for bDMARDs.

**Table 1:** Demographic data for the early RA cohort (ARCTIC cohort) and the established RA cohort (ULRABIT cohort) and in combination

	ARTIC cohort				ULRABIT cohort				Combined cohort					
Variable	*N*	Median	25 percentile	75 percentile	*N*	Median	25 percentile	75 percentile	*N*	Median	25 percentile	75 percentile	Min	Max
Age	230	53.9	41.7	62.5	212	53.2	42.05	61.45	442	53.6	42	62.1	18	86
Female sex, *n* (%)	230	141	61.3%		212	171	80.7%		442	312	70.6%			
Right hand dominance, *n* (%)	230	212	92.2%		201	198	98.5%		431	410	95.1%			
Right hand SJC, *n* (%)	162	90	55.6%		152	93	61.2%		314	183	58.3%			
Positive anti-CCP, *n* (%)	230	186	80.9%		211	164	77.7%		441	350	79.4%			
Positive RF, *n* (%)	230	164	71.3%		209	147	70.3%		439	311	70.8%			
Disease duration (months)	*n.a.*	*n.a.*	*n.a.*	*n.a.*	212	97.1	35.7	173.5	212	97.1	35.7	173.5	0.7	480
Symptom duration (days)	230	5.6	2.8	10.2	*n.a.*	*n.a.*	*n.a.*	*n.a.*	230	5.6	2.8	10.2	0	23.9
SJC28	230	6	3	11	210	5	2	10	440	5	3	10	0	26
TJC28	230	6	3	11	210	4	1	9	440	6	2	10	0	28
ESR	229	20	11	32	209	25	13	39	438	22	12	35	1	113
CRP	230	7	3	18	209	6	2	14	439	7	3	16	0.3	126
Doctors Global VAS	229	36	23	55	205	28	20	40	434	33	22	48	0	93
Patient Global VAS	230	49.5	31	70	209	50	29	73	439	50	29	71	0	100
Ultrasound scoring: Baseline														
GS sum score in right hand	225	5	2	9	212	7	4	13	437	6	3	11	0	30
GS sum score in left hand	225	5	2	8	212	6	3	12	437	6	2	10	0	30
Doppler sum score in right hand	225	3	0	7	212	3	1	8	437	3	1	7	0	26
Doppler sum score in left hand	225	2	0	5	212	3	1	7	437	2	0	6	0	25
GLOESS score in right hand	225	5	2	10	212	7	4	13	437	7	3	12	0	30
GLOESS score in left hand	225	5	2	9	212	6	3	12	437	6	2	10	0	30
Ultrasound scoring: 3 months from baseline														
GS sum score in right hand	116	0	0	2	179	5	3	11	295	3	0	8	0	28
GS sum score in left hand	116	0	0	2	179	4	2	9	295	2	0	7	0	27
Doppler sum score in right hand	116	0	0	0	179	2	0	5	295	0	0	4	0	23
Doppler sum score in left hand	116	0	0	0	179	2	0	5	295	0	0	3	0	24
GLOESS score in right hand	116	0	0	2,5	179	6	3	11	295	3	0	8	0	28
GLOESS score in left hand	116	0	0	2	179	4	2	9	295	2	0	7	0	27

*SJC* swollen joint count, *TJC* tender joint count, *ESR* erythrocyte sedimentation rate, *CRP* C-reactive protein, *VAS* visual analogue scale, *GS* grey-scale, *GLOESS* OMERACT-EULAR combined scoring system

Of the 442 patients, only 437 (99%) were included in the ultrasound evaluation as five patients from the ARTIC cohort were enrolled without an ultrasound examination at baseline whereas all 212 patients in the ULRABIT cohort had an ultrasound examination at time of inclusion. At 3 months follow-up, 295 patients had an ultrasound examination—116 patients from the ARTIC study and 179 patients from the ULRABIT study. For the whole cohort, 21 (5%) patients had left hand dominance and 410 (95%) had right hand dominance, and for 11 patients, the information was missing. Similarly, for the whole cohort, 183 (42%) patients had the right hand as the most clinically affected hand and 131 (30%) had the left hand as the most affected hand at time of treatment initiation, and for 123 (28%) patients, both hands were clinically equally affected (same number of swollen joints in both hands), and for one patient, the clinical joint information was missing.

### Differences in inflammatory activity between hands

**Fig. 1:**
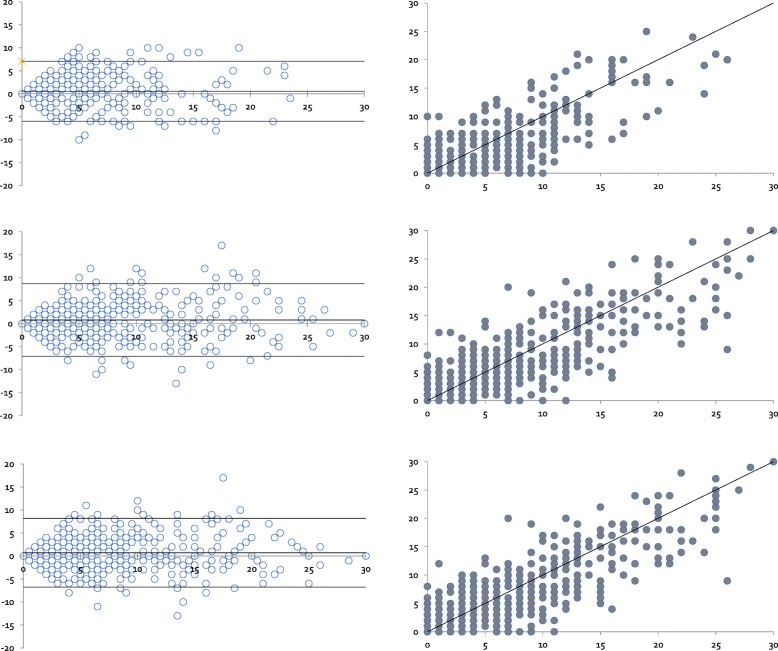
Bland-Altman plot of the distribution of the Doppler sum score, Global synovitis score (GLOESS) sum score, and grey-scale (GS) sum score for right and left hands. **a** Scatter plot: Doppler sum score ultrasound measured on the right and the left hand, with line of equality. **b** Bland-Altman plot: Difference against mean for Doppler sum score data. **c** Scatter plot: GLOESS sum score measured on the right and the left hands, with line of equality. **d** Bland-Altman plot: Difference against mean for GLOSS sum score data. **e** Scatter plot: GS sum score measured on the right and the left hands, with line of equality. **f** Bland-Altman plot: Difference against mean for GSUS sum score data

To assess if any systematic errors could potentially be found between the right- and left-hand side, Bland-Altman scatter plots were made (Fig. 1) to give a visual impression of potential differences and to assess if further analysis should be carried out. Though there seemed to be an even distribution between the right and the left hand for the GS sum score, Doppler sum score, and the GLOESS score, the right hand appeared to be potentially more involved for all three sum scores than the left.

Subsequent analyses were carried out to assess if the potential differences were present between the dominant and non-dominant hands, the right and the left hands, and the more clinically affected hand and the less clinically affected. The analyses of differences in sum scores between the dominant versus the non-dominant hand revealed that the dominant hand was *not* more inflammatory active than the non-dominant (mean difference, 95%CI) for GS sum score − 0.69 (− 1.61 to 0.24; *p* = 0.15; both confidence limits within the equivalence margin), power Doppler sum score − 0.58 (− 1.35 to 0.20; *p* = 0.14; both confidence limits within the equivalence margin), and GLOESS − 0.79 (− 1.76 to 0.18; *p* = 0.11; both confidence limits within the equivalence margin) (Table 2). Similar results were found for the right versus left hand where the mean difference for the GS sum score was − 0.75 (− 1.68 to 0.18; *p* = 0.11; both confidence limits within the equivalence margin), for the power Doppler sum score − 0.61 (− 1.38 to 0.16; *p* = 0.12; both confidence limits within the equivalence margin) and for the GLOESS − 0.89 (− 1.85 to 0.08; *p* = 0.07; both confidence limits within the equivalence margin).

**Table 2:** Ultrasound inflammatory activity in the dominant hand versus the non-dominant hand, in the hand with clinically more swollen joint versus less swollen joints, and in the right hand versus left hand using different composite scores for the early and established RA cohorts combined

Baseline				Change values from baseline to 3 months		
	GS sum score (0–30)	Doppler sum score (0–30)	GLOESS (0–30)	Δ GS sum score (0–30)	Δ Doppler sum score (0–30)	Δ GLOESS (0–30)
	Mean (95% CI)	Mean (95% CI)	Mean (95% CI)	Mean (95% CI)	Mean (95% CI)	Mean (95% CI)
*Dominant hand (*n* = 431)						
Dominant	7.75 (7.09 to 8.40)	4.81 (4.26 to 5.35)	8.07 (7.39 to 8.75)	− 3.11(− 3.65 to − 2.57)	− 2.57(− 3.10 to − 2.04)	− 3.40(− 3.98 to − 2.82)
Non-dominant	7.06 (6.41 to 7.72)	4.22 (3.68 to 4.78)	7.28 (6.60 to 7.96)	− 3.12 (− 3.66 to − 2.58)	− 2.35(− 2.88 to − 1.82)	− 3.29(− 3.87 to − 2.71)
Difference	− 0.69 (− 1.61 to 0.24)	− 0.58 (− 1.35 to 0.20)	− 0.79 (− 1.76 to 0.18)	− 0.005(− 0.76 to 0.75)	0.22(− 0.52 to 0.96)	0.11(− 0.70 to 0.92)
*P* value	0.15	0.14	0.11	0.99	0.57	0.79
*Clinical hand (*n* = 314)						
Worst clinical (more SJC)	8.51 (7.87 to 9.16)	5.37 (4.83 to 5.91)	8.83 (8.16 to 9.50)	− 3.79 (− 4.32 to − 3.25)	− 3.05(− 3.57 to − 2.52)	− 4.06(− 4.63 to − 3.49)
Least clinical (fewer SJC)	6.30 (5.65 to 6.94)	3.66 (3.13 to 4.20)	6.52 (5.85 to 7.19)	− 2.45(− 2.98 to − 1.92)	− 1.87(− 2.40 to − 1.35)	− 2.64(− 3.21 to − 2.06)
Difference	− 2.21 (− 3.12 to − 1.30)	− 1.70 (− 2.47 to − 0.94)	− 2.31 (− 3.26 to − 1.36)	1.34 (0.60 to 2.08)	1.17 (0.44 to 1.91)	1.43 (0.63 to 2.22)
*P* value	< .0001	< .0001	< .0001	0.0004	0.002	0.0005
*Hand side (*n* = 437)						
*Right side*	7.78 (7.12 to 8.44)	4.82 (4.28 to 5.37)	8.12 (7.44 to 8.80)	− 3.19(− 3.73 to − 2.65)	− 2.64(− 3.17 to − 2.11)	− 3.49(− 4.07 to − 2.91)
*Left side*	7.03 (6.37 to 7.68)	4.21 (3.6 to 4.76)	7.23 (6.55 to 7.91)	− 3.05(− 3.59 to − 2.51)	− 2.28(− 2.81 to − 1.75)	− 3.21(− 3.78 to − 2.63)
Difference	− 0.75 (− 1.68 to 0.18)	− 0.61 (− 1.38 to 0.16)	− 0.89 (− 1.85 to 0.08)	0.14(− 0.61 to 0.89)	0.36(− 0.38 to 1.10)	0.29(− 0.52 to 1.09)
*P* value	0.11	0.12	0.07	0.71	0.34	0.49

*Analyzed using a factor for the specific analyses (dominant, clinical and hand, respectively), and trial (ARCTIC and ULRABIT, respectively) as fixed effects, and the patient-ID was applied as a random effect

*SJC* swollen joint count, *Δ* delta values from baseline to 3 months, *GS* grey scale, *GLOESS* the OMERACT-EULAR combined score

When analyzing the importance of SwJ in the 314 of the 437 patients having differences in SwJ between the two sides, the analyses of the hand with more swollen joints showed a statistically significant difference in inflammatory activity as compared to the hand with less swollen joints for all three US variables: GS sum score 2.21 (1.30 to 3.12; *p* < .0001), Doppler sum score 1.70 (0.94 to 2.47; *p* < .0001), and GLOESS 2.31(1.36 to 3.26; *p* < .0001). However, with the pre-specified clinically important difference of 3 in sum scores, only the GS and GLOESS sum score may have a clinical impact whereas only a borderline clinical significance was found for the Doppler sum score as the upper confidence limit was lower than the equivalence margin of 3.

### Differences in inflammatory activity assessed over time

Clinically, there was a significant change in DAS for the ARCTIC cohort and in DAS28 (ESR) for the ULRABIT cohort from baseline to 3 months (for ARCTIC (mean (SD)) − 1.75 (0.94) and for ULRABIT − 1.08 (1.26) – *p* < 0.001 for both) [4].

Differences in (exhibiting a) change during treatment were assessed for GS sum score, Doppler sum score, and GLOESS for the dominant versus the non-dominant hand, the right versus left hand, and the clinically more affected hand versus the less clinically affected hand (Table 2) and found that the clinically more affected hand changed more than the less clinically affected hand (mean difference, 95%CI) for GS sum core 1.34 (0.60 to 2.08; *p* < 0.0004), Doppler sum score 1.17 (0.44 to 1.91, *p* < 0.002) and GLOESS 1.43 (0.63 to 2.22; *p* < 0.0005). No differences in change over time were found for the right versus left hand and the dominant versus the non-dominant hand.

## Discussion

In this agreement study comprising both early and established RA patients, we assessed differences in inflammatory activity in the right hand, the dominant hand, and the hand with clinically more swollen joints as compared to its counterpart. We found that the clinically more affected hand at baseline (determined as the hand with more clinically swollen joints) was also the more inflammatory active side—independent of using GS sum score, Doppler sum score, or the GLOESS. Similarly, the clinically most affected side was also found to be the hand exhibiting a greater change for all sum scores during treatment. These findings are to some extent in line with the subset of studies applying the unilateral 7-joint score where the most affected hand evaluated is the hand with the most swollen and/or tender joint—in these studies, this hand was sensitive to change after onset or switch of therapy (DMARDs and/or biologic) [10, 11]. In the current study, we did not evaluate the differences in inflammatory activity between hands with more and less tender joints as tender joints in contrast to swollen joints are not necessarily related to inflammatory joint involvement [21–24].

In clinical trials and in a daily clinical setting when assessing a treatment effect, it is common practice to choose the most inflamed joints for evaluation as they are more likely to exhibit a change during treatment. It was therefore of interest to evaluate if for unilateral assessment one hand was by nature always more inflamed than its counterpart based solely, i.e., on handedness as this would have impact on clinical trials and for standardized assessments in the clinic. Based on the findings in this research-on-research study applying a clinically significant cutoff of 3 in ultrasound sum scores, a default decision cannot be made on which side to scan. A clinical joint evaluation for SwJ is mandatory for choosing the optimal side for assessing treatment effect. However, the predictive value of ultrasound for treatment response and for obtaining remission has yet to be determined. Our data may however be used in subsequent prognostic factor research to explore whether the most involved hand is more appropriate (than any arbitrary decision) when trying to predict outcomes in longitudinal cohort studies and to predict who will benefit from new potential effective drugs.

As the dominant hand is often chosen in MRI studies based on reports that this hand is having more erosive changes over time [16, 17], one could speculate that this would be linked to the dominant hand being more inflammatory active than the non-dominant hand. In ultrasound studies, higher levels of inflammation in RA patients with both active disease and in remission have been shown to be related to erosive progression [25–27]; however, we were not able to show that the dominant hand was significantly more inflammatory active than the non-dominant hand nor was there any difference per se in inflammatory activity between the right and left hands. During treatment, neither the dominant hand nor the right hand displayed significantly greater change than the opposite hand.

Reduced joint combinations when using a 78-joint score as gold standard all respond well to biological treatment and all seem to give comparable information about the inflammatory activity in RA patients as a comprehensive ultrasound examination [12]. The best reduced set of joints have yet to be established [13], and though bilateral joint examinations better retain the inflammatory load of the patients than a unilateral evaluation [8], there is an interest in reducing examination time for feasibility reasons in daily clinical practice but also in clinical trials when applying ultrasound as an outcome measurement instrument. One of the often-mentioned reasons for the hampered implementation of ultrasound in the clinic is the lack of time. The current study has used the hands as a model as they are involved in all reduced joint sets [7–11], and examination solely of the hands has previously been shown to detect more than 91% of RA patients in remission with subclinical inflammation [28] and hence appears to retain valuable information about the inflammatory load of the patient.

Though much emphasis has been put on the presence of Doppler activity for assessing the degree of inflammation [3, 4], it has recently been demonstrated that also synovial hypertrophy without Doppler activity may change during treatment [29, 30], and it is therefore interesting that the most affected side for synovitis is independent of applying GS sum score or the GLOESS. The Doppler sum score showed statistically but only borderline clinical significance. This supports that both aspects of the synovitis complex (GS synovial hypertrophy and Doppler activity) are important and that if choosing unilateral scoring systems even when working with an ultrasound equipment with an insensitive Doppler, it is still the hand with the most swollen joints that should be chosen at time of evaluation and for follow-up.

In the current study, we applied a cutoff for clinically important differences in sum score of 3 for synovial hypertrophy, Doppler and GLOESS between the two hands based on expert opinion. One could argue that a lower cutoff would be equally correct, but we chose a stricter approach resulting in GS and GLOESS sum scores showing clinical and statistical differences between the hand with more and less SwJ. The Doppler sum score, though, was only borderline clinically different, but showed a statistically significant difference. However, the minimal clinically important difference in sum score for GS and Doppler has yet to be established and should be tested in subsequent studies.

The strength of the current study is the sample size and that the analyses include both early and established RA patients making the findings relevant in clinical practice. In addition, the two studies used the similar scoring system based on a US atlas. In the current datasets, 28% of the patients had similar clinical findings in both hands. In such cases, we have no recommendation regarding which side to choose for longitudinal follow-up of a unilateral ultrasound score, and the ultrasonographer may choose freely which side to scan.

## Conclusion

No hand is per default more inflamed than its counterpart. The hand with clinically more swollen joints is probably the best choice for monitoring ultrasound activity in clinical practice and in trials and thus the best indicator for the side to choose if a unilateral scoring system is used.

## Additional file

Additional file 1: Statistical analysis plan. (PDF 419 kb)

## Data Availability

Please contact the authors for data requests.
