# Supplementary material for: Assessing synovitis in the hands in patients with rheumatoid arthritis by ultrasound: an agreement study exploring the most inflammatory active side from two Norwegian trials
Source: Arthritis Res Ther. 2019 Jul 5;21:166. doi: 10.1186/s13075-019-1930-y (PMC6612125; doi:10.1186/s13075-019-1930-y)
Supplement: Supplementary file 1 — Statistical analysis plan. (PDF 419 kb) [file 13075_2019_1930_MOESM1_ESM.pdf]

Statistical Analysis Plan (SAP)

Unilateral ultrasound scoring methods for synovitis in patients with rheumatoid arthritis: An agreement study exploring the most active side

2017-dec-06

Authors/Collaborators

Lene Terslev[1,2], Robin Christensen[2], Anna-Birgitte Aga[3], Espen A. Haavardsholm[3,4], Hilde B. Hammer[3]

Affiliations

[1]Center for Rheumatology and Spine Diseases, Rigshospitalet, Copenhagen, Denmark

[2]Musculoskeletal Statistics Unit, The Parker Institute, Bispebjerg and Frederiksberg Hospital, Denmark

[3]Department of Rheumatology, Diakonhjemmet Hospital, Oslo, Norway

[4] Dept. of Health Management and Health Economics, University of Oslo, Oslo, Norway

Correspondence to:

Dr. Lene Terslev, MD, PhD

Center for Rheumatology and Spine Diseases, Rigshospitalet, Copenhagen

Nordre Ringvej 57; DK-2600 Glostrup; Fax: 38 63 39 61

INTRODUCTION

Background

Ultrasound has been validated as an outcome measurement instrument for rheumatoid arthritis (RA) and has been demonstrated to be able to detect changes over time during treatment. Ultrasound may detect changes in both grey-scale and Doppler and are usually scored separately using a semi-quantitative scoring system (0-3) indicating the grade of severity of the pathology found.

The Doppler activity is reflecting the hyperaemia of the inflammatory process and is believed to represent inflammatory changes whereas grey-scale changes may also reflect chronic changes. Recently, a new consensus-based combined scoring system taking both components into account has been published.

In order to increase feasibility several reduced joint sets for scoring synovitis have been proposed over the last years ranging from 6 – 12 joints evaluating synovitis either unilaterally or bilaterally aiming at maintaining as much of the inflammation seen in a more elaborate joint evaluation (32 – 78 joints).

Rationale

If a unilateral scoring system should be applied, there is currently no guidance on how to choose "target side" at time of inclusion (e.g., if part of a randomised controlled trial). In magnetic resonance imaging (MRI), where only one side can be evaluated, the dominant hand is often chosen but to the best of our knowledge there is no evidence indicating that the dominant hand is more affected than the non-dominant hand.

Aim/hypotheses

The primary aim is to evaluate if the dominant side is the preferred side to be chosen for unilateral scoring systems in RA patients, as judged by clinically significantly more Doppler signal at baseline, as well as judged from the change observed at the usual 3 months assessment.

Secondary aim is to investigate if the right hand is more inflammatory active than the left hand and thirdly to evaluate if the hand with most swollen joints is more active than the hands with less swollen joints.

METHODS

Study design

This study is designed as an agreement study which explores the impact on ultrasound in a cross-sectional sample as well as evaluated based on the observed changes at three months from baseline. Results of reliability and agreement studies are intended to provide information about the amount of error inherent in any diagnosis, score, or measurement. While reliability is usually defined as the ratio of variability between participants (e.g., patients) - and thus describe our ability to differentiate between patients, agreement is the degree to which ratings can be considered identical. When two methods are compared neither provides an unequivocally correct measurement, so we try to assess the degree of agreement.

In order to assess the agreement between ultrasound joint inflammation scores, assessed either on the dominant vs the non-dominant hand, we will re-analyse two independent ultrasound datasets from Norway that have been collected bilaterally at baseline.

Datasets: Participants and sample size

Baseline and three months follow-up assessments from two RA (trial) cohorts will be included: an early RA cohort (ARCTIC trial) and an established RA cohort (ULRABIT trial) with patients initiating DMARD treatment; csDMARD in the early cohort and initiating or switching bDMARD in the established cohort. The ARCTIC (early RA) was registered in the ClinicalTrials.gov database (NCT01581294) and the ULRABIT trial (established RA) was registered in the Anzctr.org.au

database (ACTRN12610000284066). Both cohorts have previously been assessed and published in studies for evaluating treatment response and determining reduced joint sets for monitoring.

From the ARCTIC trial we anticipate that we will be able to include 238 patients (recruited between September 2010 and April 2013), and from the ULRABIT trial (recruited from 2010 to June 2013), 212 patients with established RA will be available.

Ultrasonography assessments

The hands were used as model evaluating MCP 1-5, PIP 2+3 and wrist (DRUJ, RCJ, ICJ). In the original trials (ARCTIC and ULRABIT) an extensive US examination was performed by experienced sonographers using 0–3 semiquantitative scoring systems for both GSUS and PDUS in each of the following 36 joints and four tendons: meatacarpophalangeal (MCP) 1–5, proximal interphalangeal (PIP) 2–3, radiocarpal, intercarpal, distal radioulnar, elbow, knee, talocrural, and metatarsophalangeal (MTP) 1–5 bilaterally.

The range of the sum scores was 0–120 for both GSUS and PDUS and the scanning protocol was a slight modification of a previously published 32-joint protocol (with addition of bilateral PIP2–3, ECU and TP tendons) with the same probe placement and patient positioning and with an US atlas as a reference. All the sonographers in the multicentre study (ARCTIC) underwent training in the form of an US workshop with both static and dynamic hands-on exercises to calibrate readers, and the workshop was repeated yearly. This validation study showed high inter- and intra-observer reliability, and most of the examiners in this study were also examiners in the multicentre study.

Siemens Antares Sonoline machines (SiemensMedical solutions, Mountain view, California, USA) with linear probes (5–13MHz and setting at 11.4MHz) and identical settings optimised for PDUS in superficial joints (pulse repetition frequency (PRF) 391 Hz, low wall filter and frequency 7.3MHz), or GE Logiq E9 (GE Medical Systems Ultrasound and Primary Care Diagnostics, Wauwatose, Wisconsin, USA), were used in all the 11 hospitals for the ultrasonography assessments. The US machines were calibrated and optimised for PD sensitivity using technical personnel from the manufactures, to ensure correct settings in all machines.

In the single-centre study (ULRABIT) all US examinations were performed by the same sonographer who has previously demonstrated high inter- and intra-observer reliability for scoring synsovitis in joints.

A Siemens Antares Sonoline machine (SiemensMedical solutions, Mountain view, California, USA) with a linear probe (5–13MHz and setting at 11.4MHz) and identical settings optimised for PDUS in superficial joints (pulse repetition frequency (PRF) 391 Hz, low wall filter and frequency 7.3MHz) were used.

Laboratory and clinical examinations

Assessments included erythrocyte sedimentation rate (ESR), C-reactive protein (CRP, mg/L), anti-cyclic citrullinated Peptide (anti-CCP) and 0–100 mm visual analogue scales (VAS) for physician's and patient's global assessments of disease activity for both trials (ARCTIC and ULRABIT). For the early RA cohort, 44 swollen joint count (44 SJC) and Ritchie Articular Index were performed, while 28 swollen and tender joint counts (28 SJC and 28 TJC, respectively) were performed in the established RA cohort. The Disease Activity Score (DAS) was calculated in the early RA cohort, whereas the DAS28 was calculated in the patients with established RA, both scores based on ESR.

Outcomes and analyses

The primary outcome measure will be the Doppler sum score (PDUS, ranging from 0 to 30), whereas the Global Synovitis Score (GLOSS; range 0-30), and Grey-Scale sum score (GSUS; range 0-30) will be considered key secondary outcome measures.

The primary analyses are

Differences in inflammatory activity between the dominant and non-dominant hand (Doppler sum score $\geq$ 3) assessed by both the

- Doppler sum score (PDUS: 0 – 30)
- Global score (GLOSS: 0-30)

- Grey scale sum score(GSUS: 0-30)

Secondary analyses include

- Differences in inflammatory activity between the hand with most clinically swollen joints and the hand with less swollen joints
- Differences in inflammatory activity between the right and left hand *per se*.

Tertiary analyses include

Confirmatory approach: These will be based on all of the above based on a cohort (change from baseline) data set, where the sensitivity to change will be the outcome of interest.

Stratified analyses

- We will perform stratified analyses to explore whether the outcome vary with the RA state; we will compare the Early vs. Established RA Cohort for the above mentioned outcomes.

Statistical methods

In an agreement study, it is most unlikely that different US methods (e.g. Dominant vs Non-Dominant) will agree exactly, by giving the identical result for all individuals. Thus, we want to know by how much the methods are likely to differ; if this is not enough to cause problems in clinical interpretation we can decide on using any of the two (i.e., the two can be used interchangeably).

If the dominant and non-dominant sides are unlikely to give readings which differ by more than, say, 3 units (on a 0-30 sum score), we could use either measure interchangeably. On

the other hand, if the result from ultrasound scans differ by significantly more than 3 units (on a 0-30 sum score), the one with the highest signal will likely be the preferred choice.

The first step will be to plot the data and draw the line of equality on which all points would lie if the two (PDUS) meters gave exactly the same reading every time. This will help the eye in gauging the degree of agreement between measurements, though, the Bland-Altman Plot might be more informative. The second step will be the Bland-Altman Plot. A plot of the difference between the bilateral measures their mean will be more informative (right vs left hand), as it would display considerable lack of agreement between the measures. The plot of difference against mean also allows us to investigate any possible relationship between the measurement error and the true value. Obviously we do not know the true value, and for that exact reason the mean of the two measurements is the best estimate we have.

In an equivalence trial, we would conclude that two treatments are equivalent if the observable difference (MD) between them lies within an established interval for predefined clinical equivalence margin, (−d, d). We will define a reasonable equivalence margin in this study, to be a 95% Confidence Interval around the observed paired mean difference: -2.99 to +2.99. We will test the similarity between measures using SAS for Mixed Models (PROC MIXED). We will analyse and report the (least squares) mean values (continuous outcomes) and the difference between them; the model will include a factor for the specific analysis, and trial (ARCTIC and ULRABIT, respectively) as a fixed effect, and the patient-ID will be applied as a random effect.

RESULTS: Anticipated outline

Figure 1

Flow diagram of the progress through the phases of randomised trials: that is, enrolment, intervention allocation, follow-up, and the present data analysis (agreement study).

Table 1: Baseline demographics and patient characteristics*

Variable	ARCTIC trial: n=238 (early RA)	ULRABIT trial: n=212 (established RA)	Total: n=450 RA patients
Age, years, mean (SD)			
Female sex, n (%)			
Right hand dominance, n (%)			
Positive anti-CCP, n (%)			
Positive RF, n (%)			
Symptom duration, months, median (25–75 percentile)			
Twenty-eight-swollen joint count, median (25–75 percentile)			
Twenty-eight-tender joint count, median (25–75 percentile)			
ESR, mm/h, median (25–75 percentile)			
CRP, mg/L, median (25–75 percentile)			
Investigator's global assessment VAS, mean (SD)			
Patient's global assessment VAS, mean (SD)			
Number of previously used synthetic DMARDs, mean (SD)			
Number of previously used biological DMARDs, mean (SD)			
Ultrasound score (combined):<br>- PDUS (0-30)<br>- GLOSS (0-30)<br>- GSUS (0-30)			

*Descriptive statistics will be applied depending on the nature (distribution) of the data.

Figure 2

2A: Scatter Plot: Doppler sum score (PDUS) ultrasound measured on the right vs. left hand side; with line of equality.
2B: Bland & Altman Plot: Difference against mean for Doppler sum score (PDUS) data.
2C: Scatter Plot: GLOSS sum score measured on the right vs. left hand side; with line of equality.
2D: Bland & Altman Plot: Difference against mean for GLOSS sum score data.
2E: Scatter Plot: GSUS sum score measured on the right vs. left hand side; with line of equality.
2F: Bland & Altman Plot: Difference against mean for GSUS sum score data.

Table 2: Ultrasound inflammatory activity according to different analysis scenarios*

Analysis	**Doppler sum score (0-30)**	**GLOSS (0-30)**	**GSUS (0-30)**
Dominant Hand			
Dominant:	Mean ± SE	Mean ± SE	Mean ± SE
Non-dominant:	Mean ± SE	Mean ± SE	Mean ± SE
Difference:	MD (95%CI; P-value)	MD (95%CI; P-value)	MD (95%CI; P-value)
Clinical Hand			
Worst swollen:	Mean ± SE	Mean ± SE	Mean ± SE
Least swollen:	Mean ± SE	Mean ± SE	Mean ± SE
Difference:	MD (95%CI; P-value)	MD (95%CI; P-value)	MD (95%CI; P-value)
Handedness (side)			
Right:	Mean ± SE	Mean ± SE	Mean ± SE
Left:	Mean ± SE	Mean ± SE	Mean ± SE
Difference:	MD (95%CI; P-value)	MD (95%CI; P-value)	MD (95%CI; P-value)
Dominant Hand (adjusted)*			
Yes:	Mean ± SE	Mean ± SE	Mean ± SE
No:	Mean ± SE	Mean ± SE	Mean ± SE
Difference:	MD (95%CI; P-value)	MD (95%CI; P-value)	MD (95%CI; P-value)

*Mixed Model: Analysis of Covariance (ANCOVA) model will be used to analyse mean values (continuous outcomes); the model will include a factor for the specific analysis scenario, and trial (ARCTIC and ULRABIT, respectively) as a fixed effect; PtID will be applied as a random effect.

**Based on the same model(*) plus sex, age, disease duration, and DAS28 as extra covariates.
